# Antitumor potential of new low molecular weight antioxidative preparations from the white rot fungus *Cerrena unicolor* against human colon cancer cells

**DOI:** 10.1038/s41598-018-37947-z

**Published:** 2019-02-13

**Authors:** Anna Matuszewska, Dawid Stefaniuk, Magdalena Jaszek, Mateusz Pięt, Adrian Zając, Łukasz Matuszewski, Iga Cios, Marcin Grąz, Roman Paduch, Renata Bancerz

**Affiliations:** 1 Department of Biochemistry, Maria Curie-Skłodowska University, Lublin, Poland; 2 Department of Virology and Immunology, Maria Curie-Skłodowska University, Lublin, Poland; 3 Department of Comparative Anatomy and Anthropology, Maria Curie-Skłodowska University, Lublin, Poland; 4 Department of Paediatric Orthopaedics and Rehabilitation, Medical University of Lublin, Lublin, Poland; 5 Department of General Ophthalmology, Medical University of Lublin, Lublin, Poland

## Abstract

The aim of this study was to investigate the anticancer and antioxidant activities of low molecular weight subfractions isolated from secondary metabolites produced by the wood degrading fungus *Cerrena unicolor*. Human colon cancer cells (stage I) HT-29 and human normal colon epithelial cells CCD 841 CoTr were used in the research. The present study demonstrated that the low molecular weight subfractions exhibited inhibitory activity towards human colon cancer cells HT-29 at a concentration range of 25–200 μg/mL. All 6 subfractions inhibited proliferation of cells down to 47.5–9.2% at the highest concentrations in a dose-dependent manner. The most desired activity was exhibited by subfractions S, 3, 4, and 5, as the proliferation of HT-29 cells was inhibited to the greatest extent (16.5, 47.5, 42.7, and 26.1% of the control, respectively), while the effect on CCD 841 CoTr cells was the mildest (inhibition to 54.4, 71.4, 79.4, and 53.4%, compared to the control, respectively). The microscopic observation revealed that all extracts induced programmed cell death, i.e. apoptosis (up to 44.4% (subfraction 6) towards HT-29 and less than 20% (most fractions) towards CCD 841 CoTr), with no or a significantly low level of necrosis in both cell lines at the same time.

## Introduction

Mushrooms offer a wide range of benefits associated with consumption thereof and, therefore, they are regarded as functional foods^1^. The health-enhancing properties of edible mushrooms have been analyzed for years, especially in the traditional Eastern medicine and in the folk medicine of the West. They are characterized by high contents of easily digestible protein, fatty acids with a favorable ratio of polyunsaturated to saturated acids, vitamins mainly from the B group and vitamins A, E, and C, as well as polysaccharides, including dietary fiber^2^. The bioactive substances produced by fungi can be divided into two main groups: low molecular compounds, e.g. terpenoids or phenolic compounds, and high molecular weight compounds, e.g. polysaccharides and enzymes^3^. Both groups of compounds exhibit biological activities such as antioxidant, anticancer, immunostimulating, antiatherosclerotic, neuroprotective, anti-inflammatory, antiallergic, antibacterial, antiviral, or hypoglycemic effects. The range of these activities results from the diversity of bioactive components present in fungi, such as proteins, e.g. immunomodulatory mushroom proteins (FIPS), lectins, glycoproteins, polysaccharides, phenolic compounds, indole compounds, terpenoids, or lipids (including ergosterol and its derivatives)^4–8^. The need for finding new compounds with effective antitumor properties and high selectivity towards cancer cells and low toxicity to normal cells prompts investigations of a wide variety of chemically and structurally related compounds. Therefore, the last few decades have been a period of the most intense research on substances isolated from fungi, both fruiting bodies and *in vitro* cultivated mushroom biomass. The largest group of compounds with anti-inflammatory properties is constituted by fungal terpenoids. They also exhibit antitumor, antiviral, antimalarial, anti-yeast, and antibacterial activity^9^. Monoterpenoids act cytotoxically against cervical carcinoma (HeLa) and liver cancer (HepG2) cells^10^. Diterpenoids and triterpenoids isolated from *Ganoderma lucidum* (Reishii) and *Ganoderma orbiforme*, such as ganoboninketals AG, numerous ganoderic acids, lucidadiol, luciden acids, and many others that exert a cytotoxic effect on tumor cells, have anti-malarial and anti-tuberculosis activity^9,11^. In addition, *G. lucidum* triterpenes inhibit angiogenesis, which is important in the development of tumor mass and metastasis^12^. Bisabolol produced by *Inonotus rickii* inhibits the development of myeloid leukemia as well as breast, colorectal, lung, and liver cancers^13^, and sesquiterpenoids isolated from *Pleurotus cornucopiae* are characterized by cytotoxicity to cervical cancer cells (HeLa) and liver cancer cells^14^. Phenols are another important group of low-molecular compounds of fungal origin. They have a wide range of bioactive properties; hence, they are used in the treatment of cancer, cardiovascular diseases as cardioprotective and vasodilator compounds, and agents in the treatment of brain disorders and degenerative diseases. Many of them have been found to have anti-inflammatory and anti-allergic effects^15,16^. The largest group among phenolic compounds of fungal origin comprises phenolic acids and polyphenols, for instance flavonoids, stilbene, lignans, tannins, and oxidized polyphenols. Phenolic acids are the basic phenolic compounds produced by fungi. Phenolic compounds from *Pleurotus ostreatus*, *Lentinula edodes*, or *Hypsizygus tessellatus* have antibacterial properties as well^17^. However, phenolic compounds occurring in *Hericium erinaceus* additionally induce the synthesis of a factor that activates the growth and development of nerves^18^. Another equally interesting group of fungal compounds is organic acids. These substances are compounds with antioxidant properties; therefore, they can be used in the treatment of many diseases related to oxidative stress^19^.

Colon cancer is one of the most common cancers worldwide and the second most common cancer in developed countries^20^. Surgery and chemotherapy are the routine treatments for colon cancer patients. However, the benefits of chemotherapy are still under discussion, because a large proportion of patients die due to tumor recurrence after such therapy^21^. Therefore, constant search for substances that would be effective in treating this type of cancer is crucial. An interesting fungal species, tested primarily as a very efficient source of extracellular laccase produced in non-induced growth conditions, is *Cerrena unicolor*. In addition to the laccase, it produces many other low molecular weight secondary metabolites with a broad spectrum of biological activity, which was confirmed in our previous work^22–25^. Studies carried out to date have been focused on the total low molecular subfraction obtained directly as a by-product of the production of biotechnologically important enzymes, e.g. laccase. From the point of view of possible biomedical applications, it was important to characterize and separate the preparation obtained from the culture fluid of *C. unicolor*, which was used for determination of its qualitative composition as well as biological properties (especially the anticancer effect).

## Methods

### Strain, medium, growth processing, and separation of fungal samples

*Cerrena unicolor* (Bull. ex Fr.) Murr. was obtained from the culture collection of Regensburg University and deposited in the fungal collection of the Department of Biochemistry (Maria Curie-Sklodowska University, Poland) under strain number 139 (ITS sequence deposited in the GenBank under accession number DQ056858)^26^. The fermentor scale cultivation was performed according to the procedure described in earlier studies^24^. The post-culture fluid obtained from *C. unicolor* fungus culture was used in the study. The mycelium was separated from the culture fluid, which was subsequently subjected to preliminary separation on an ultrafiltration cartridge with 10 kDa MWCO (EMD Millipore™ Prep/Scale Spiral-Wound Ultrafiltration Modules: TFF-2), yielding two fractions with a molecular weight below 10 kDa and above 10 kDa. The lower mass fraction (ex-LMS) was further concentrated using Reverse Osmosis Membrane TFC-75F (Aquafilter Inc. USA) and subjected to further analysis. The fraction with a molecular weight below 10 kDa was fractionated on a chromatography column 5.0 × 30 cm (diameter × length) packed with Sephadex G-15. As a result of the separation, two low-molecular fractions were obtained - a fraction with a molecular mass above 1.5 kDa and a fraction corresponding to a mass below 1.5 kDa (subfraction S), which was lyophilized and used for further fractionation on a 2.5 × 120 cm Sephadex G-15 column. The resulting 6 subfractions (1, 2, 3, 4, 5, 6) were lyophilized and characterized. Fractions with the highest therapeutic potential were further fractionated on an Agilent Infinity 1260 chromatograph, using a 250 × 3 mm Knauer column packed with Eurospher II 100-3 C18A and equilibrated with 0.1% TFA in water. The 5-µl sample was eluted for 40 min at 0.1 ml/min with a 0.1% TFA solution in water and subjected to 30-min gradient separation: 0–50% water with 0.1% TFA and acetonitrile with 0.1% TFA at a 0.5 ml/min flow rate. Detection was conducted at 214 nm. The peaks with intensity above 200 µAu from 20 runs were collected, lyophilized, and analyzed with FTIR spectroscopy.

### Cell cultures

The research was conducted on two cell lines: human normal colon epithelial cells CCD 841 CoTr (ATCC No. CRL-1807) and human colon cancer cells (stage I) HT-29 (ATCC No. HTB-38). The cells were cultured in RPMI 1640 medium (HT-29) or a mixture of DMEM and RPMI 1640 media (1:1)(CCD 841 CoTr), supplemented with 10% (v/v) FBS and antibiotics (100 U/mL penicillin, 100 μg/mL streptomycin), and kept at 37 °C (HT-29) or 34 °C (CCD 841 CoTr) in humidified atmosphere with 5% CO_2_.

### Preparation of samples

Freeze-dried extracts were dissolved in RPMI medium to obtain a stock solution of 4 mg/mL. Subsequently, the extracts were dissolved in RPMI or RPMI:DMEM (1:1) with 2% (v/v) FBS to obtain desired concentrations.

### Determination of proteins, carbohydrates, and phenolic compounds

Protein concentrations were determined using the Bradford reagent and bovine serum albumin as a standard^27^. The total content of the phenolic compounds was determined with diazosulfanilamide using the DASA test^28^, where absorbance was measured at 500 nm and vanillic acid was used as a standard. The total carbohydrate content was determined with the phenol-sulfuric acid assay using D-glucose as a standard^29^.

### Antioxidant properties

#### DPPH free radical-scavenging test

The total antioxidant capacity of the sub-fractions was determined using the DPPH radical as a reagent, according to the procedure described by Paduch *et al*.^30^. This method is based on the ability of 1,1-diphenyl-2-picrylhydrazyl (DPPH) to decolorize in the presence of antioxidants. Subsequently, 100 μL of the test compound at concentrations ranging from 6.25 to 800 µg/mL were mixed with 100 μL of the DPPH solution (0.2 mg/mL in ethanol) and absorbance at 515 nm was determined after 2, 5, 10, 15, 20, and 30 min of incubation at room temperature. Trolox, i.e. the well-known standard with strong antioxidant activities, was used as a positive control. The results where expressed as trolox equivalents in mM per gram of dry analyte.

#### ABTS radical-scavenging test

The ABTS radical-scavenging activities of the fractions were determined according to the method proposed by van den Berg *et al*.^31^, Duo-Chuan^32^, and Re *et al*.^33^ with modification. The stock solution was prepared by dissolving 7.4 mM ABTS and 2.6 mM potassium persulfate in MQ water. After 16 h, the concentrated ABTS stock solution was diluted with phosphate buffered saline (PBS) pH 7.4 to absorbance recorded at 734 nm. Subsequently, 10 μL of the test compound at concentrations ranging from 6.25 to 800 μg/mL were mixed with 990 μL of the ABTS radical solution and absorbance was measured. The results where expressed as trolox equivalents in mM per gram of dry analyte.

### Anticancer assay

#### MTT method

The method is based on the ability of mitochondrial succinate dehydrogenase to reduce yellow tetrazolium salt (MTT) to purple formazan crystals. Such activity is performed by live cells; therefore, absorbance is directly proportional to the quantity of live cells^34,35^.

After 96- h incubation of the cells in 96-well plates (100 μL of 1 × 10^5^ cells/mL) with the studied extracts, 25 μL of 5 mg/mL MTT (Sigma) were added to each well. After 3 h, 100 μL of 10% SDS (Sigma) in 0.01 M HCl (POCH) were added and incubated for 24 h. The plates were read using a Microplate Reader and absorbance at 570 nm was measured (Molecular Devices Corporation; Menlo Park, CA, USA).

#### Apoptosis evaluation – Hoechst 33342/PI staining

Evaluation of apoptosis and necrosis in the cell cultures was performed by staining with DNA-intercalating fluorochromes – Hoechst 33342 (Sigma) and propidium iodide (PI) (Sigma)^36^. The morphological analysis was performed under a confocal microscope Axiovert 200 M (Zeiss, Jena, Germany) with a scanning head module LSM 5 PASCAL. Cells exhibiting blue fluorescence of nuclei (fragmented or with condensed chromatin) were interpreted as early apoptotic. Morphologically similar cells with pink fluorescence were defined as late apoptotic. Cells with pink fluorescence of whole nuclei were classified as necrotic.

The cultures were grown in Petri dishes (3 cm diameter); 2 mL of 1 × 10^5^ cells/mL were poured to each dish. After 24 h incubation with the extracts, 5 μL of a Hoechst 33342 (0.4 mg/mL) and propidium iodide (0.5 mg/mL) mixture (2:3 ratio) were added. The samples were incubated for 5 min and then observed under a fluorescence microscope. At least 1000 cells in randomly selected areas were counted for each sample and the ratio of apoptosis and necrosis was calculated.

#### Wound assay

The cells were poured onto Petri dishes (3 cm diameter) and cultured until formation of a monolayer. Afterwards, wounds were made, the cells were washed twice with PBS, and fresh media with or without (control) the studied fractions were added. The cells were incubated for 24 h and stained according to the May-Grünwald-Giemsa method. One dish was stained immediately after wounding – the wound control. The images were taken under Olympus BX51 (Olympus Optical Co. Ltd, Japan) with an Olympus SC30 head module. The distance between the faces of migrating cells was measured using CellSans software and calculated in comparison to the control regarded as 100%.

#### Zymography

After the incubation with the studied extracts at 100 μg/mL, the supernatants above the cell cultures were collected. 20 μL of the supernatants were loaded on a 10% polyacrylamide gel with addition of 0.1% gelatin (POCH) and 0.001% SDS (Sigma). Electrophoresis was performed at 120 V (starting at 90 V, until the face reached the separating gel). Afterwards, the gels were washed twice (2 × 15 min) in renaturing buffer (2.5% Triton X-100 (Sigma) in 50 mM Tris-HCl (Sigma) followed by two 15-min washes in 50 mM Tris-HCl buffer. Subsequently, the gels were incubated in incubation buffer (5 mM CaCl_2_ in 50 mM Tris-HCl) at 37 °C for 24 h. After the incubation, the gels were stained with 0.15% Coomassie Brilliant Blue R-250 dissolved in methanol: acetic acid: glycerol: water (16:2:1:23, by vol.) for 30 min. and destained in methanol: acetic acid: glycerol: water (8:2:1:29, by vol.)^37–39^. Densitometric analysis was performed using Image Studio Lite software (LI-COR Biosciences).

### FT-IR Spectroscopy Analysis of ex-LMS Samples

The analyses of ex-LMS subfractions were carried out using lyophilizates. FTIR spectroscopy was performed with a spectrometer (Thermo Scientific Nicolet 8700 A with FT Raman Nicolet NXR module) in the wavelength range 4000–400 cm^−1^.

### Statistical analysis

All analyses were performed in at least 3 replications and the data were analyzed using GraphPad Prism ver. 5.01. The results are presented as mean ±  SD. Statistical significance was evaluated with the one-way Anova test and post-hoc Dunnett’s test (all columns compared to the control).

## Results

Our previous studies demonstrated that the extracellular fraction of secondary metabolites of less than 10 kDa had cytotoxic, antioxidant, and antimicrobial effects; therefore, further fractionation was performed in order to examine the individual subfractions^22–25^. In this study, we focused on selected properties of extracellular secondary metabolites with a molecular mass below 1.5 kDa. The separations of the starting fractions based on the molecular weight of their compounds yielded six separate formulations (S,1–6) differing in their qualitative composition and biological properties.

### Characterization of the main biochemical properties of the extracellular low molecular subfractions

The analysis of the chemical composition of the obtained subfractions (1–6) showed the presence of protein (except for subfraction 3), sugars, and phenolic compounds (Table 1). The highest concentration of low molecular weight peptides was recorded for fraction 6 (31.8 μg/mL): it was about 10 times higher than for fraction 1–5. The highest concentration of total carbohydrates was recorded for fractions 4 and 5 (404.4 and 328.7 μg/mL, respectively). The concentration of phenolic compounds was similar in all the analyzed subfractions.

**Table 1:** Chemical composition of the extracellular *C. unicolor* ≤ 1.5 kDa subfraction of low molecular weight metabolites (S) and its subfractions (1–6); yield of total carbohydrates, protein content, and concentration of phenolic compounds.

Subfraction	S	1	2	3	4	5	6
Protein (μg/mL)	10.9 ± 1.8	8.0 ± 0,4	1.9 ± 0.1	0 ± 0.0	2.3 ± 0.1	3.7 ± 0.2	31.8 ± 0.5
Total carbohydrates (μg/mL)	220.2 ± 2.8	253.8 ± 31.4	287.2 ± 44.1	215.5 ± 11.1	404.4 ± 30.5	328.7 ± 33.6	190.4 ± 44.2
Total phenolic compounds (μg/mL)	55.4 ± 1.3	64.7 ± 0.2	50.5 ± 1.5	57.9 ± 1.2	61.5 ± 2.1	57.8 ± 1.1	73.5 ± 1.1

### Antioxidant properties of the extracellular low molecular subfractions

The antioxidant capacity of the subfractions prepared was evaluated in the study (Table 2). All subfractions showed antioxidant activity, but the highest values were recorded for subfraction 6: these were 569.8 mM/g for the ABTS method and 168.4 mM/g for the DPPH method. The weakest antioxidant properties were found for the fraction corresponding to a mass below 1.5 kDa (subfraction S) and subfraction 3.

**Table 2:** Radical scavenging effects of the extracellular ≤ 1.5 kDa subfraction of low molecular weight metabolites (S) from *C. unicolor* and its subfractions (1–6) assessed with the ABTS and DPPH radical-scavenging method and expressed as trolox equivalents [mM/g].

Subfraction	S	1	2	3	4	5	6
ABTS radical-scavenging	176.2 ± 0.4	308.8 ± 1.1	243.1 ± 0.1	169.0 ± 0.1	239.3 ± 1.0	303.0 ± 0.6	569.8 ± 3.8
DPPH radical-scavenging	52.0 ± 0.7	117.7 ± 7.2	119.5 ± 5.3	0 ± 0.2	91.5 ± 5.1	119.6 ± 4.3	168.4 ± 3.2

Data are means  ±  SD of three measurements (n = 3).

### Inhibition of proliferation

**Figure 1:**
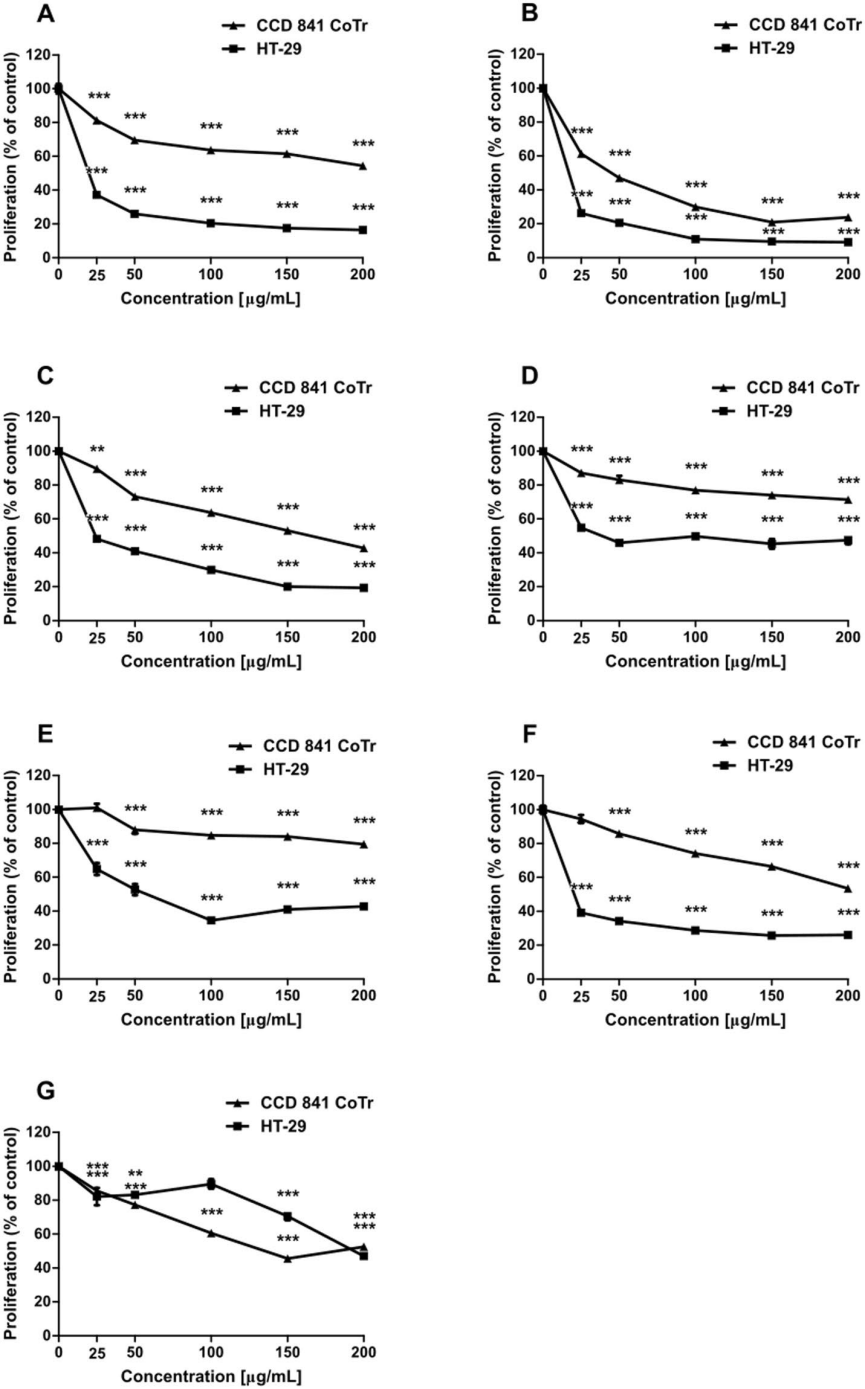
Inhibition of proliferation of CCD 841 CoTr and HT-29 cells by fractions S (**A**), 1 (**B**), 2 (**C**), 3 (**D**), 4 (**E**), 5 (**F**), and 6 (**G**) measured with the MTT method. Values are expressed as a percentage of the control regarded as 100%; *p < 0.01, **p < 0.005, ***p < 0.001, one-way Anova, Dunnett’s test.

All subfractions inhibited cell proliferation in a dose-dependent manner. Furthermore, except for 6, the activity was stronger towards cancer cells, as the proliferation of the HT-29 cells was reduced to 16.5, 9.2, 19.4, 47.5, 42.7, and 26.2% of the control by fractions S, 1, 2, 3, 4, and 5 at 200 μg/mL, respectively (Fig. 1A–F). Subfraction 6 at concentrations of 25 μg/mL and 200 μg/mL had a stronger effect on the cancer cells (proliferation reduced to 82.2% and 47.1% of the control, respectively) than on the normal cells (proliferation inhibited to 85.6% and 52.6%, compared to the control, respectively) (Fig. 1G). In turn, at the concentrations of 50, 100, and 150 μg/mL, a greater effect was exerted on the CCD 841 CoTr cells. The most desired activity was exhibited by subfractions S, 3, 4, and 5, i.e. the proliferation of the HT-29 cells was inhibited to the greatest extent, while their effect on the CCD 841 CoTr cells was the mildest (Fig. 1A, D–F).

### Induction of apoptosis

**Figure 2:**
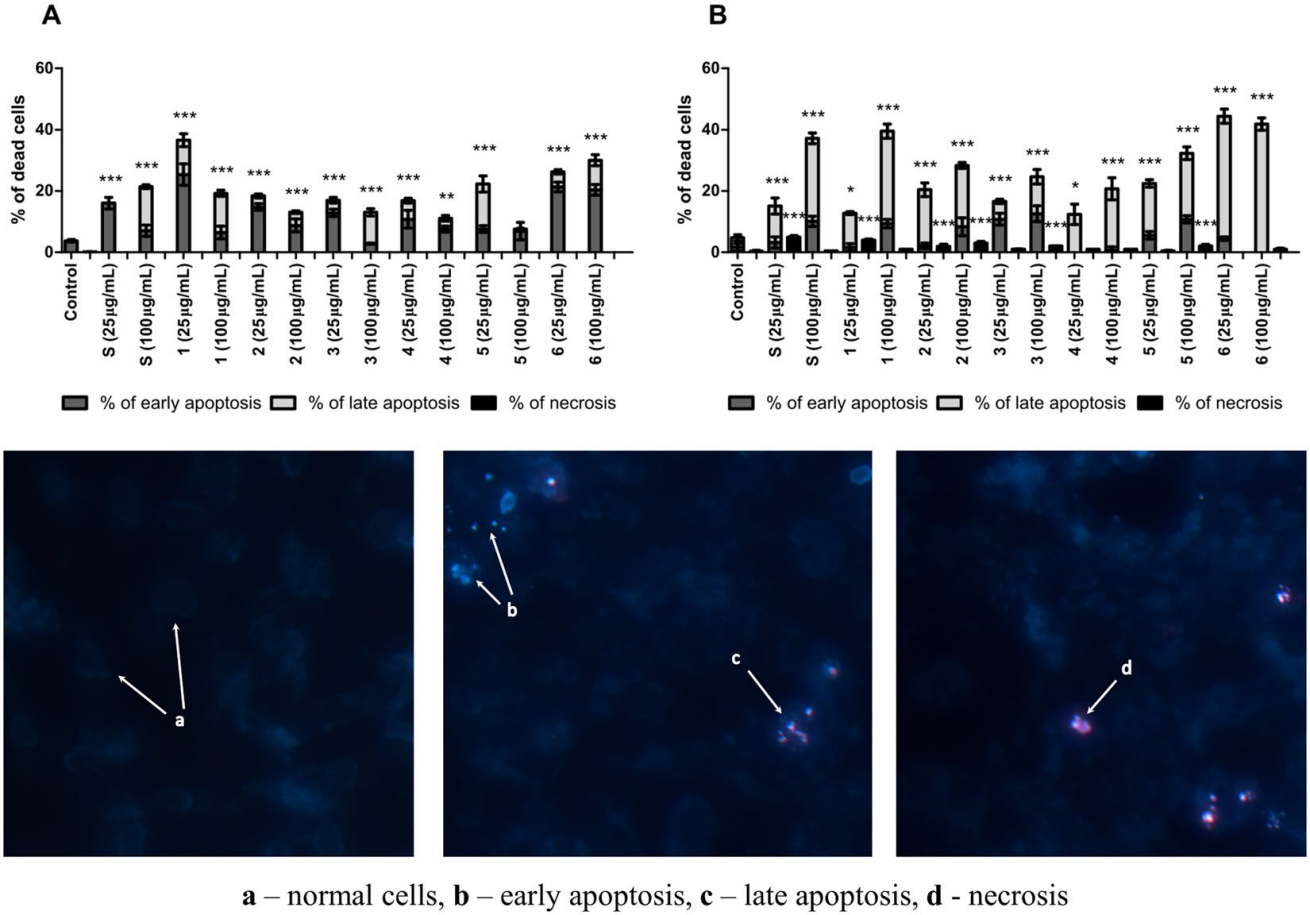
Ratio of apoptosis (early and late) and necrosis in CCD 841 CoTr (**A**) and HT-29 (**B**) cells induced by the studied fractions (upper panel) and representation of particular effects in cells: (a) normal cells, no effect, (b) early apoptosis, (c) late apoptosis, and (d) necrosis.

The microscopic observations revealed that all extracts induced programmed cell death (PCD), i.e. apoptosis, with no or a significantly low level of necrosis in both cell lines at the same time. There was a correlation between the apoptosis phase (early/late) and the cell line. Early apoptosis was dominant in the CCD 841 CoTr cell line, while the late phase dominated in HT-29. Among the extracts at 25 μg/mL, the highest level of programmed cell death in HT-29 was induced by subfraction 6 (44.4%) (Fig. 2B), while the rate of apoptosis in CCD 841 CoTr was significantly lower (26.2%) (Fig. 2A). In turn, at 100 μg/mL, the highest level of PCD was also induced by subfraction 6 in HT-29 (41.9%) and in CCD 841 CoTr (30.1%) (Fig. 2). All fractions at 100 μg/mL exhibited significantly greater pro-apoptotic activity in the HT-29 cell line, compared to the level of PCD in CCD 841 CoTr. All results were compared to the control in both cell lines. The control exhibited a significantly low level of apoptosis and necrosis (3.8% and 4.7% of apoptosis in CCD 841 CoTr and HT-29 cells, respectively, and 0.3% and 0.4% of necrosis in CCD 841 CoTr and HT-29 cells, respectively). The highest pro-apoptotic activity towards HT-29 cells was exhibited by subfractions S, 1, 5, and 6. All variants, except for 1 and 4 at 25 μg/mL, exerted a stronger pro-apoptotic effect on cancer cells than on normal cells.

### Inhibition of migration

**Figure 3:**
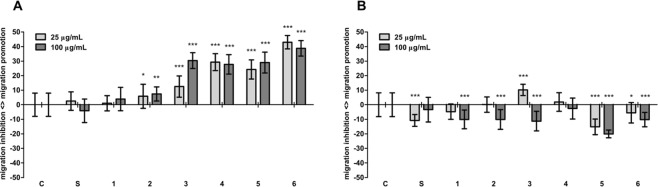
Effect of the fractions on the migration of CCD 841 CoTr (**A**) and HT-29 cells (**B**) (wound assay); *p < 0.01, **p < 0.005, ***p < 0.001, one-way Anova, Dunnett’s test.

**Figure 4:**
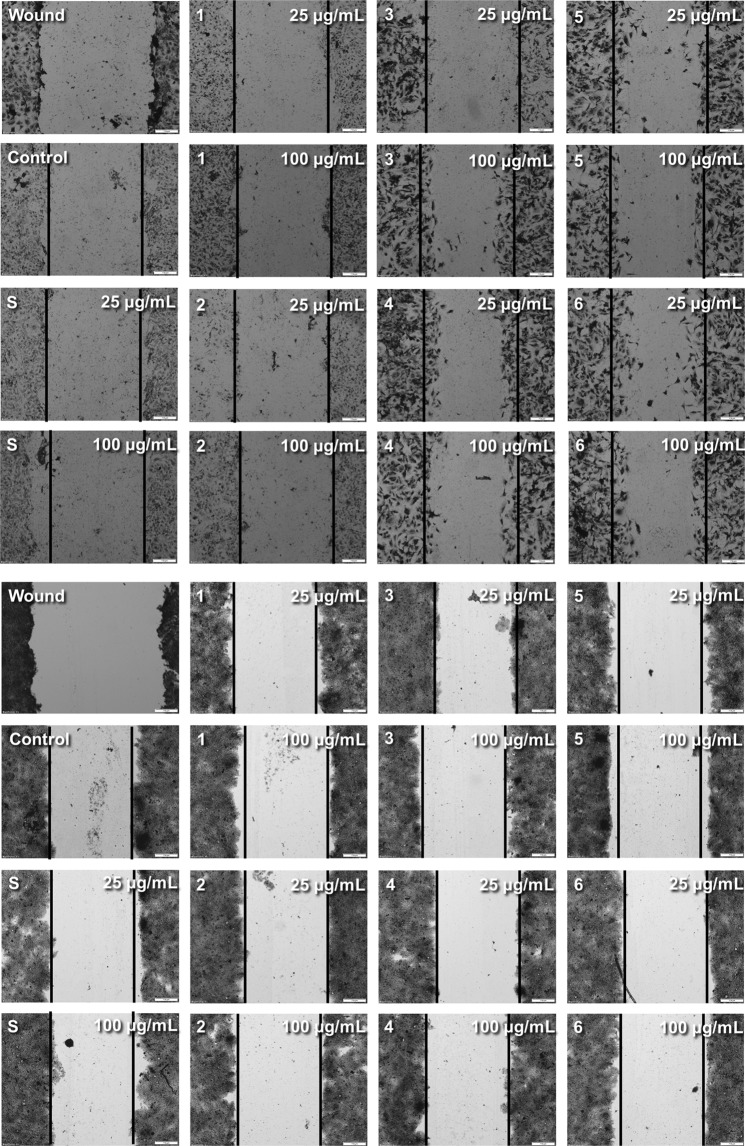
Effect of the fractions on the migration of CCD 841 CoTr (upper panel) and HT-29 (bottom panel) cells – wound assay. The width of the control wound is indicated with lines in each picture. The measurement bar indicates 200 μm.

Inhibition of cancer cell migration may indicate anti-metastatic properties. Except for subfraction 3 and 4 at 25 μg/mL, all subfractions inhibited migration of cancer cells by 2.5 and 3.4% (crude subfraction S), 4.8 and 10.1% (subfraction 1), 0.1 and 10.1% (subfraction 2), 11.2% (subfraction 3), 2.6% (subfraction 4), 15.2 and 20.1% (subfraction 5), and 5.5 and 10.3% (subfraction 6), compared to the control, at 25 and 100 μg/mL, respectively (Figs. 3B and 4 bottom panel). In contrast, all subfractions promoted migration of normal cells (Figs. 3A and 4 upper panel). Although subfraction S at 100 μg/mL inhibited migration of normal cells, the effect was mild (4.1% of inhibition, compared to the control) and not statistically significant. The most potent activity was exhibited by subfractions 5 and 6. They inhibited migration of HT-29 cells and promoted migration of CCD 841 CoTr cells to the greatest extent. The effect on cancer cells did not exceed 20% of inhibition, but it must be noted that the HT-29 cells originated from stage I cancer.

### Zymography

**Figure 5:**
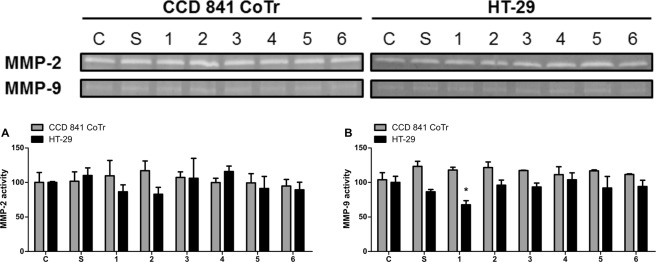
Activity of MMP-2 and MMP-9 secreted by CCD 841 CoTr and HT-29 cells incubated with the studied extracts at 100 μg/mL evaluated with zymography; the activity was measured densitometrically and presented in comparison to the control (regarded as 100%); *p < 0.01, **p < 0.005, ***p < 0.001, one-way Anova, Dunnett’s test.

In a majority of cases, the zymographic analysis revealed no statistically significant influence of the subfractions on the activity of matrix metalloproteinases (MMPs) 2 and 9 secreted by the cells (Fig. 5). Despite the evident changes in the MMP activity in each variant, only subfraction 1 exerted a statistically significant effect towards the HT-29 cells, i.e. a decrease in the MMP-9 activity to 67.6% of the control (Fig. 5B).

After analysis of the zymography gels, another band with molecular mass <50 kDa was observed. The densitometric analysis revealed that all subfractions promoted the activity of the protease with this particular mass secreted by CCD 841 CoTr. The strongest effect was exerted by subfraction 6 (to 128.9%, compared to the control) (Supplementary, Figs. S1A and S2), and it was the only statistically significant result in the case of normal cells. These results correspond with the migration assay, where the strongest migration induction of CCD 841 CoTr was exhibited by subfraction 6 (Fig. 3A). The activity of this protease secreted by the HT-29 cells was inhibited by subfractions 2, 3, 4, 5, and 6, but only subfraction 4 had a statistically significant effect, i.e. a decrease in the activity to 40.9% of the control.

### FT-IR Spectroscopy Analysis

Fraction 5 was selected for the FT-IR analysis due to its antiproliferative effect on the HT-29 cells and at the same time the weakest effect on the normal cell line (CCD 841 CoTr) out of the studied fractions. The comparison of the FT-IR spectra of the HPLC-purified subfraction 5 components against the Pharmaceuticals, Drugs and Antibiotics Spectra database St. JAPAN indicates the presence of compounds derived from hydroquinone, resorcinol, salicylaldehyde, and p-methoxyphenol.

## Discussion

Colon cancer is the fourth most commonly diagnosed and the third most lethal cancer in the world. Despite intense efforts to develop treatments, effective measures are still not available. Therefore, extracts from natural products are still a very promising source of new anticancer drugs. Mushrooms are known as one of the most productive groups of useful natural products, including anticancer compounds. White rot fungi release a broad spectrum of low-molecular metabolites to the substrate, which not only support the decomposition of lignocellulosic material, but also have interesting health-promoting properties. In previous studies, we have shown that *C. unicolor* is an interesting fungus due to the unique properties of the laccase produced by this species as well as the interesting properties of extracellular secondary metabolites, which are a source of a waste product generated during the preparation of enzymes^22–25,40^.

Our previous analyses showed that the scavenging ability of the extracellular low molecular weight preparation was the same or even higher than that of the model antioxidant substances, i.e. trolox and ascorbic acid. The activity of this fraction determined using DPPH in the concentration range of 6.25–800 μg/mL ranged from 10% to 59% of free radical scavenging, but was at the level of 20% to 90% in an ABTS test^22^. In this study, we have also found that the six new low molecular weight preparations from *C. unicolor* have antioxidant properties. The antioxidative properties of some fungal species were also identified by other authors using the same determination methods. Kim *et al*. showed that edible fungi *Pleurotus ostreatus, Agaricus bisporus*, *Pleurotus eryngii*, and *Lentinus edodes* and medicinal mushrooms *Agaricus blazei, Phellinus linteus, Ganoderma lucidum*, and *Inonotus obliquus* had antioxidant activity and free radical scavenging ability, as demonstrated by the DPPH assay^11,41^. The white rot fungus belongs to the group of medical fungi characterized by slightly higher antioxidant activity, compared to edible fungi^41^. Other authors have described isolation of polyphenols with antioxidant properties from *Inonotus xeranticus* and *Phellinus linteus*^42^ as well as *Ramaria flava*^10^. The antioxidant profile of the studied preparations is closely related to the content of phenolic compounds and -OH groups of carbohydrates. Subfraction 6 showed the highest concentration of phenolic compounds among all the studied subfractions (73.5 μg/mL) and had the best antioxidant properties at the same time. To the best of our knowledge, these are the first studies showing that the byproduct obtained during isolation of laccase from the *C. unicolor* fungus is such an interesting source of antiproliferative substances in relation to colon cancer cells. Our results showed that apoptosis was gradually enhanced with an increase in the concentration of the subfractions. All fractions at 100 μg/mL exhibited significantly greater pro-apoptotic activity in the HT-29 cell line in relation to the level of PCD in CCD 841 CoTr. Similar results were obtained by Lim *et al*., who studied the effect of hispidin isolated from *P. linteus* on colon cancer cells, i.e., CMT-93 and HCT 116. They reported anticancer activities of the phenolic compound hispidin from the medicinal fungus *P. linteus*, which were reflected in induction of apoptosis in colon cancer cells. They found that hispidin inhibited cell viability in a dose-dependent manner^43^. Lee *et al*. reported anticancer activities of cordycepin isolated from *Cordyceps militaris* through induction of apoptosis in human HT-29 colon cancer cells^44^. Doskocil *et al*. examined mycelial extracts for cytotoxic properties against colon cancer cell line HT-29 and human Caco-2 colon adenoma cells. White rot fungi *Daedalea quercina, Merulius tremellosus, Piptoporus betulinus, Pycnoporus cinnabarinus, Lentinula edodes*, and *Ganoderma lucidum* showed cytotoxicity towards HT-29 tumor cells expressed as EC_50_ of 60, 186, 73, 31, and 302.115 μg/mL, respectively^45^. In their studies, these authors used isolated substances from mycelia or full ethanol extracts obtained from the mycelium of selected species, while our research was conducted on the hitherto untested culture fluid. In this work, we also demonstrated that, except for subfractions 3 and 4, the other subfractions inhibited the migration of cancer cells, favoring the migration of normal cells, which may indicate anti-metastatic properties. Therefore, the studied preparations may prevent invasion of adjacent tissues by cancer cells at early stages and prevent metastasis before the cells acquire the ability to move and release from the primary tumor to reach distant niches.

Metalloproteinases of the extracellular matrix (MMPs) and their tissue inhibitors play an important role in the process of colon carcinogenesis. One of the metalloproteinases with significant importance in the development of colon cancer is MMP-9^46^. Colon cancer cells have been shown to be able to synthesize metalloproteinases, including MMP-9, a proteolytic enzyme capable of digesting type IV collagen - the main component of the basal membrane. The degradation of type IV collagen by MMP-9 is found in both invasion and metastasis^47^. Similarly, Wu *et al*. showed that the expression of MMP-2 and MMP-9 was significantly higher in colon cancer tissues^48^. The zymographic analyses carried out in this work showed some effect of the subfractions on the activity of matrix metalloproteinase (MMP) 9 secreted by the cells. The studied subfractions promoted the migration of the normal cells, while inhibiting the process in the HT-29 cells. Furthermore, subfraction 1 exhibited strong inhibition of the activity of MMP-9 secreted by the HT-29 cells. The results suggest that inhibition or promotion of migration of both CCD 841 CoTr and HT-29 cells may be dependent on the activity of MMPs, as well as other factors engaged in cancer progression. Furthermore, another band with molecular weight ~50 kDa was observed. The activity of this protease corresponded to the migration assay. A protease with such molecular mass may be another MMP (i.e. MMP-14) regulated by MMP-2^49,50^ or another form of MMP-9. Active MMP-9 may appear in the form of 82 kDa, but also ~65 or ~50 kDa truncated forms. It seems that low-mass forms of MMP-9 are resistant to TIMP-1 inhibition, thus the decrease in the activity of low-mass MMP by the studied subfractions is a desired result^51,52^.

The FT-IR analysis of subfraction 5, which acted pro-apoptotically on the tumor cells and affected the normal cells to a smallest extent, performed in the last stage of the study showed the presence of compounds that were relatively similar in their spectra to hydroquinone, resorcinol, salicylaldehyde, and p-methoxyphenol, e.g. phenolic substances with antioxidant activity. This result confirms previous analyses of the presence of phenolic compounds and their antioxidant properties.

To sum up the presented results, it can be concluded that the preparations isolated from the LMS fraction produced by *C. unicolor* can induce apoptosis in human HT-29 colon cancer cells and prevent metastasis. These facts suggest that these substances, obtained and characterized for the first time in this work, should be further evaluated as potential therapeutic and/or preventive agents in human colon cancer.

## Supplementary information

Gelatin zymography analysis of enzymatic activity of matrix metalloproteinases MMP-2, MMP-9 and ~50kDa MMP
