# Supplementary material for: Antitumor potential of new low molecular weight antioxidative preparations from the white rot fungus Cerrena unicolor against human colon cancer cells
Source: Sci Rep. 2019 Feb 13;9:1975. doi: 10.1038/s41598-018-37947-z (PMC6374373; doi:10.1038/s41598-018-37947-z)
Supplement: Supplementary file 1 — Gelatin zymography analysis of enzymatic activity of matrix metalloproteinases MMP-2, MMP-9 and ~50kDa MMP [file 41598_2018_37947_MOESM1_ESM.pdf]

Antitumor potential of new low molecular weight antioxidative preparations from the white rot fungus *Cerrena unicolor* against human colon cancer cells

Anna Matuszewska[1], Dawid Stefaniuk[1], Magdalena Jaszek[1], Mateusz Pięt[2], Adrian Zając[3], Łukasz Matuszewski[4], Iga Cios[1], Marcin Grąz[1], Roman Paduch[5], Renata Bancerz[1]

[1]Department of Biochemistry, Maria Curie-Skłodowska University, Lublin, Poland

[2]Department of Virology and Immunology, Maria Curie-Skłodowska University, Lublin, Poland

[3]Department of Comparative Anatomy and Anthropology, Maria Curie-Skłodowska University, Lublin, Poland

[4]Department of Paediatric Orthopaedics and Rehabilitation, Medical University of Lublin, Poland

[5]Department of General Ophthalmology, Medical University of Lublin, Lublin, Poland

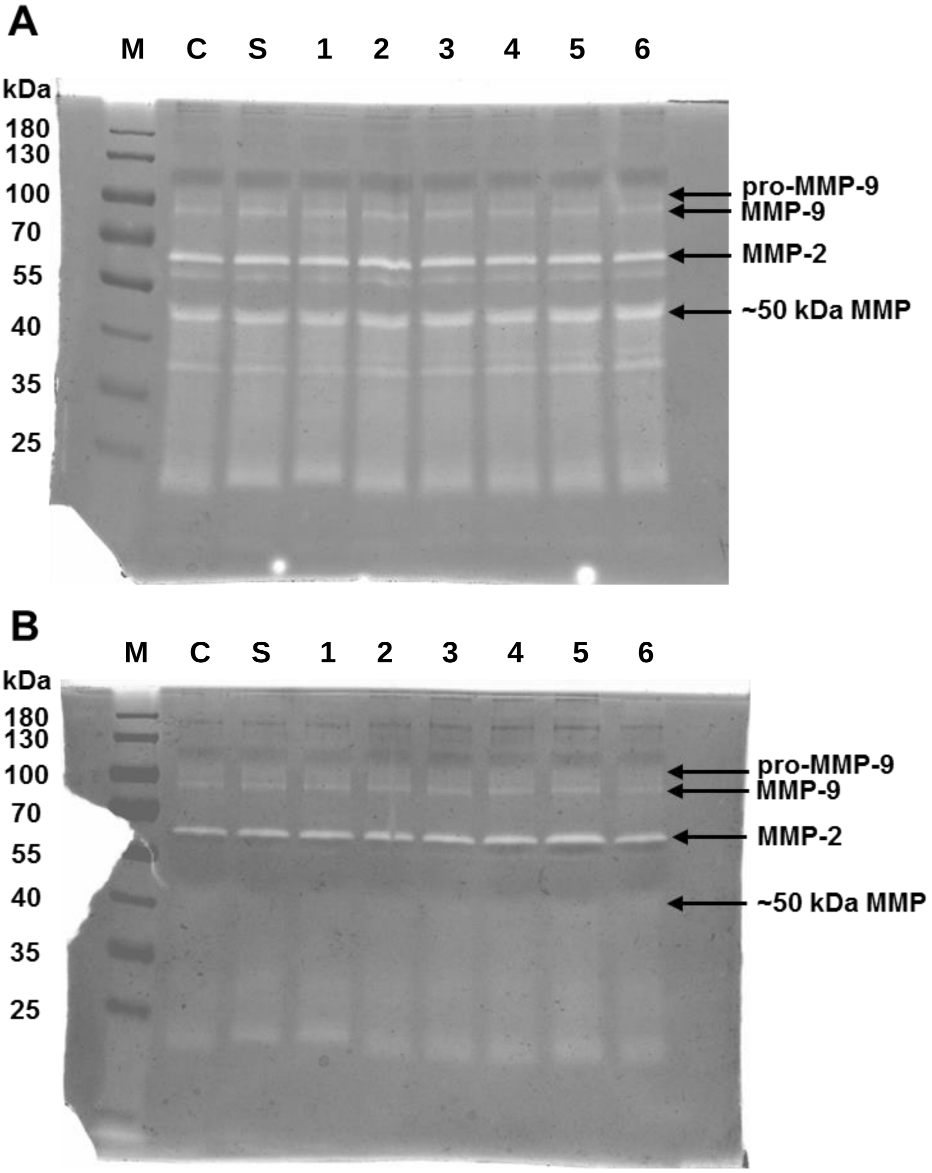

Fig. S1. Gelatin zymography analysis of enzymatic activity of matrix metalloproteinases MMP-2 and MMP-9 secreted by CCD 841 CoTr (A) and HT-29 (B) cells treated with fractions S and 1 - 6; M – molecular mass marker; C – control.

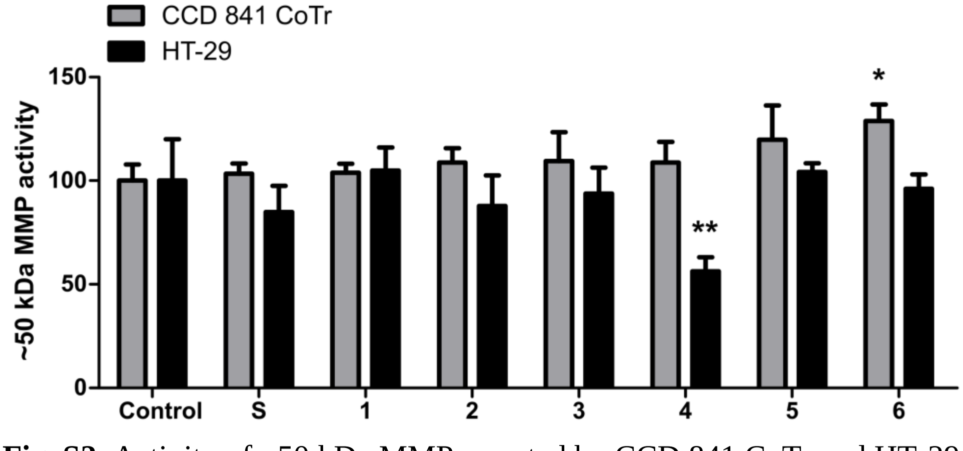

Fig. S2. Activity of ~50 kDa MMP secreted by CCD 841 CoTr and HT-29 cells.
